# Clinical efficacy and learning curve of posterior percutaneous endoscopic cervical laminoforaminotomy for patients with cervical spondylotic radiculopathy

**DOI:** 10.1097/MD.0000000000030401

**Published:** 2022-09-09

**Authors:** Ran Yao, Ming Yan, Qingchen Liang, Hongqing Wang, Zuyao Liu, Fu Li, Hao Zhang, Ke Li, Fenglong Sun

**Affiliations:** a The No.2 Department of Orthopedics, Beijing Rehabilitation Hospital Affiliated to Capital Medical University, Beijing, China; b Department of Spinal Surgery, First Hospital of Bethune, Jilin University, Changchun, China.

**Keywords:** cervical spondylotic radiculopathy, cumulative sum analysis, learning curve, minimally invasive, posterior percutaneous endoscopic cervical laminoforaminotomy, surgical procedures

## Abstract

In this study, we aimed to investigate the clinical efficacy and learning curve of posterior percutaneous endoscopic cervical laminoforaminotomy (PPECLF) in patients with cervical spondylotic radiculopathy (CSR). A total of 64 patients with CSR received PPECLF. Clinical outcome scores included the visual analog scale, Japanese Orthopedic Association score, neck disability index, and modified Macnab criteria. Radiological outcomes included the disc height, C2 to C7 Cobb angle, and range of motion. The learning curve was evaluated using cumulative sum analysis. Patients were divided into accumulation phase and mastery phase groups (A and B), and general data and surgical efficacy were compared between the 2 groups. Follow-up ranged from 12 to 24 months. Clinical outcome scores improved significantly at the final follow-up, and there were no differences in radiological outcomes. Surgical efficacy was excellent and good in 82.8% of patients. The operative time showed a decreasing trend with the accumulation of cases. Patients were divided and the 26th case was the cutoff point according to the learning curve. No significant differences were found in the clinical outcomes between the 2 groups. Decompression with PPECLF was safe and effective in the treatment of CSR. With the accumulation of cases, the operative time was gradually shortened, and the clinical efficacy was significant. The PPECLF procedure can be performed efficiently and safely to treat CSR.

## 1. Introduction

Cervical spondylotic radiculopathy (CSR), specified by Ando in 1952, has been described as the most common type of cervical spondylosis.^[1]^ Decompression surgery is often a necessary intervention in patients with poor efficacy after conservative treatment or worsening symptoms.^[2–4]^ Anterior cervical discectomy and fusion (ACDF) was once the mainstream surgery for the treatment of cervical degenerative diseases and was regarded as the standard operation for CSR.^[5]^ However, ACDF is suspected to cause postoperative loss of disc height, adjacent segment disease (ASD), pseudarthrosis, and approach-related complications.^[6]^ The traditional posterior cervical open surgical approach causes muscle dissection, substantial trauma, high blood loss, reduced postoperative effects, and reduced patient satisfaction.^[7,8]^

Since 2019, we have performed posterior percutaneous endoscopic cervical laminoforaminotomy (PPECLF) based on the anatomy and operating path of traditional open approach surgery. A minimally invasive working channel is used to replace the long incision exposure, and proprietary endoscopic tools are used for accurate decompression. It has the same therapeutic effect as open approach surgery and maintains a high level of security.^[9,10]^ However, there are few reports on laminae decompression and nerve root canal under percutaneous endoscopic procedures.^[11–13]^ The presence of technical barriers, potential replication, and the ability to judge the proficiency of this new technology are crucial for beginners. Therefore, the aim of this study was to investigate the clinical efficacy and learning curve of PPECLF in patients with single-segment CSR.

## 2. Methods

### 2.1. Study design and patient selection

A total of 64 patients (29 men, 45%; 35 women, 55%) who experienced CSR and underwent PPECLF at our institute between March 2019 and March 2021 were retrospectively reviewed. The sample size of this study was based on that of previous studies.^[14]^ Written informed consent was obtained from all patients for the acquisition of their medical information. This study was approved by our institutional review board. The study included perioperative data and latest postoperative follow-up (FU) outcomes. Inclusion and exclusion criteria are listed in Table 1.

**Table 1:** Inclusion and exclusion criteria of PPECLF for CSR.

Inclusion criteria	Exclusion criteria
Level C3–C7	Multisegmental lesions
Single-segment lateral cervical disc herniation and/or foraminal stenosis proved by CT and MRI	Neck and shoulder pain without obvious upper limb radiating pain
Clinical symptoms of radiculopathy including severe upper limb radiating pain with/without neck and shoulder discomfort, or upper limb numbness and myasthenia	Cervical spondylotic myelopathy, severe ossification of the posterior longitudinal ligament, obvious cervical instability, or angulation and slip
Failed conservative treatment (physical therapy, medicine, neck brace, etc.) for at least 3 months	Central disc herniation or anterior osteophytes
Treated with PPECLF, and postoperative rehabilitation exercise	Previous operation history of neck, infection, fracture, tumor, serious mental disease

CSR = cervical spondylotic radiculopathy, CT = computed tomography, MRI = magnetic resonance imaging, PPECLF = posterior percutaneous endoscopic cervical laminoforaminotomy.

### 2.2. Surgical technique

**Figure 1.**
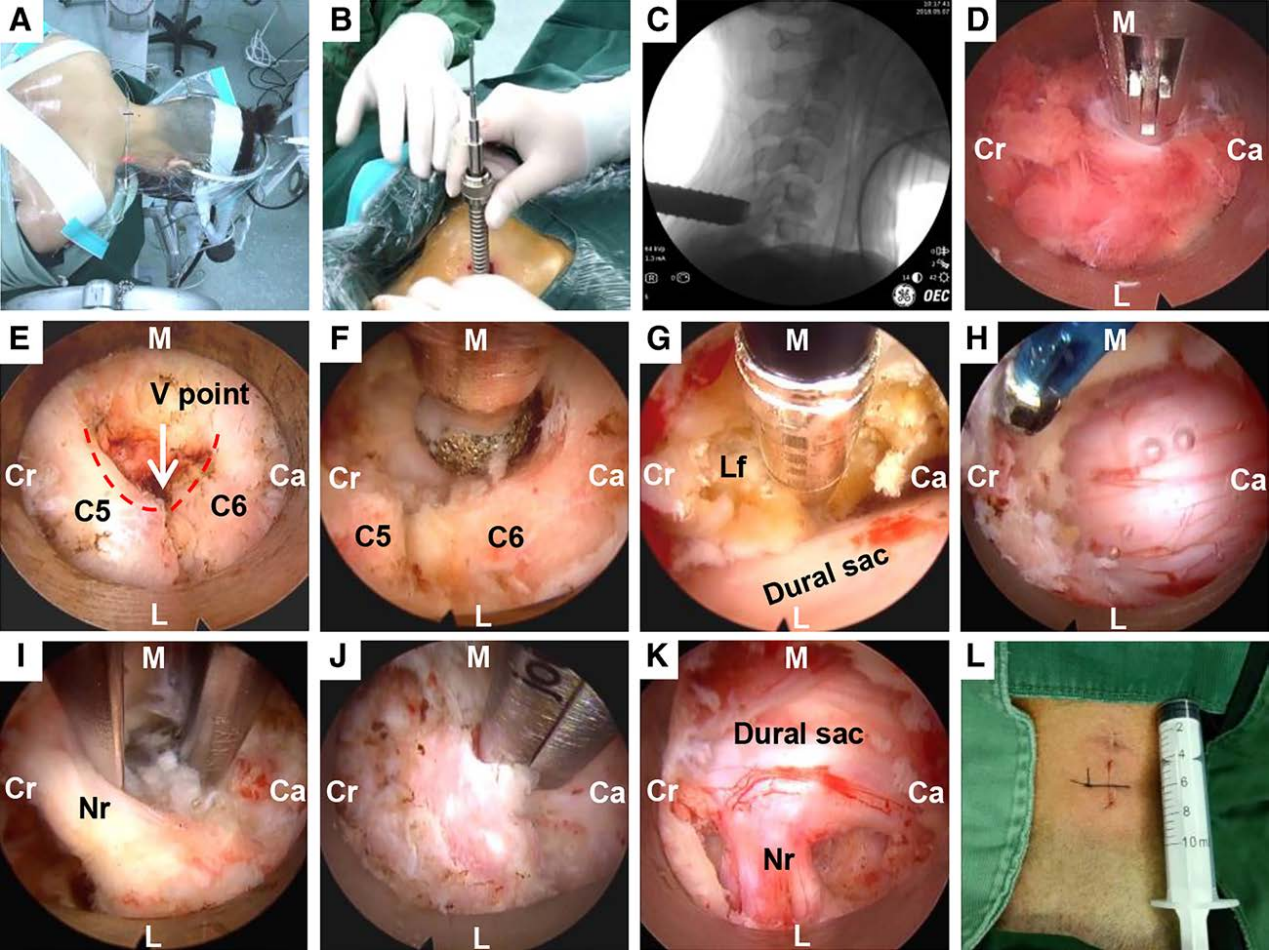
Intraoperative pictures for PPECLF. The position of patient (A). Insert the endoscopic system after the progressive expansion of the cannula (B). Confirmed the position of channel (C). Cleaned the surface of lamina and joint (D). Exposed the V point (white arrow) (E). Grinding at V point to form a window hole (F). Removed part of the ligamentum flavum (G). Coagulated the veins on the dura mater, bone surface for prehemostasis (H). Removed the nucleus pulposus tissues and osteophytes (I). Enlarged the nerve root canal for foraminotomy (J). Confirmed the nerve root was relaxed and the dural sac pulsed well (K). Postoperative surgical incision (L). Ca = caudal, Cr = cranial sides, L = lateral, M = medial, Nr = nerve root, PPECLF = posterior percutaneous endoscopic cervical laminoforaminotomy.

All surgeries were performed by a single senior spine surgeon. The iLESSYS Delta system (Joimax GmbH, Germany) was used exclusively in this study. After general anesthesia, the patient was placed in the prone position, and the head was fixed on a Mayfield traction rack (Fig. 1A). After the “C” arm X-ray machine was used to locate the target interspinal interstice, 18-gauge spinal needle puncture was performed in the gap between the lamina and facet joint (V point). A 7.0-mm skin incision was made ≈1.0 cm away from the posterior median line. A sequence of dilators were inserted (Fig. 1B, C), and a 6.0-mm working channel (outer diameter 13.5 mm and inner diameter 11.0 mm) and endoscopic system (length, 125 mm; outer diameter, 10.0 mm; inner diameter, 6.0 mm; visual field angle, 80°; visual direction angle, 30°) were placed. The operation was performed under continuous irrigation with 0.9% saline solution. The soft tissue on the surface of the lamina and facet joint was cleaned with a coagulator, and the upper and lower laminae of the responsible segments were confirmed (Fig. 1D). A 3-mm diamond abrasor was used to successively grind the upper and lower laminae at the V point, to remove the superior outer layer of the lamina and cancellous bone (Fig. 1E). The lower lamina was removed using Endo-Kerrison punches, and a 10.0-mm window hole was perforated at the V point (Fig. 1F). Then, the margin of the ligamentum flavum was revealed (Fig. 1G). After part of the ligamentum flavum was separated and excised, the spinal cord and nerve roots were identified and separated laterally (Supplemental Video File, Supplemental Digital Content, http://links.lww.com/MD/H194). The responsible nerve root was tracked until exposure. Vessels on the surface of the dura mater, bone, and tissues around the hole were coagulated for prehemostasis (Fig. 1H). Nucleus pulposus forceps were used to remove the prominent annulus fibrosus and nucleus pulposus (Fig. 1I), and diamond abrasors were used to remove proliferous osteophytes. The broken soft tissue and deep disc that could not be reached with forceps were cleaned via radiofrequency ablation. Decompression of the vertebral pedicle was performed cephalically and caudally. For nerve root canal stenosis, Kerrison punches were used to perform the foraminotomy (Fig. 1J). Finally, a nerve probe was used to explore the shoulder and underarm of the nerve root to ensure that no disc tissue remained, the nerve root was relaxed, and there was good pulsation of the dural sac (Fig. 1K).

### 2.3. Evaluation of clinical efficacy

Perioperative indicators of the patients were recorded, including operation time, amount of bleeding, complications, postoperative hospitalization day, hospitalization cost, and number of reoperations. The therapeutic effect was assessed using the visual analog scale (VAS), Japanese Orthopedic Association (JOA) score, neck disability index (NDI), and modified Macnab criteria.^[15–18]^ The radiographic outcomes of the 64 patients were compared according to preoperative and postoperative radiography and computed tomography results. Evaluation indexes included disc height, Cobb angle, and cervical range of motion (ROM).

### 2.4. Evaluation of the learning curve

A bivariate regression curve was used to reflect the variation trend of the operation time with the increase in case number. *R*^*2*^, y, and × of the model represent the fitting effect, operation time, and number of cases, respectively. The learning curve was assessed by evaluating the operation time using cumulative sum (CUSUM) analysis and the formula $\text{CUSUM}=\sum_{i=1}^{n}\left(T_{i}-M\right)$, in which *T*_*i*_ represents the individual operation time, *M* represents the mean operation time, and *n* represents the case number. The CUSUM results were plotted graphically. SPSS software (version 22.0; SPSS, Chicago, IL, USA) was used to fit the curve and obtain the equation, and *P* < .05 was considered successful fitting. The curve with the largest *R*^*2*^ was selected as the optimal model. On the CUSUM learning curve, the point of decline was the cutoff point where the later sample data were below the average. The corresponding abscissa value was the minimum number of cases required to pass the accumulation stage.^[19]^ Based on the cutoff point, patients were divided into accumulation (group A) and mastery (group B) phase groups, and the general information and clinical efficacy were compared between the 2 groups.

### 2.5. Statistical analysis

SPSS software (version 22.0; SPSS, Chicago, IL, USA) was used for statistical analysis. After the cohort data were determined to conform to a normal distribution, the mean ± standard deviation was used to represent the measurement data. Categorical variables were analyzed using the chi-square test. An independent sample Student *t* test was used for quantitative variable measurement between the 2 groups. A *P* value < 0.05 denoted statistical significance.

## 3. Results

### 3.1. Evaluation of overall efficacy outcomes

All surgeries were successful and none were converted to open surgery. The mean FU of all cases was 17.6 ± 2.8 months (range, 12–24 months). The mean operation time was 84.4 ± 28.2 min (range, 43–145 minutes). The mean intraoperative blood loss was 16.2 ± 4.1 mL (range, 5–35 mL). The mean postoperative hospitalization time was 13.5 ± 5.4 days (range, 2–45 days). Cervical JOA scores improved from 14.8 ± 1.1 preoperatively to 16.7 ± 1.2 at the last FU (*P* < .05). Neck and shoulder pain and upper limb VAS scores decreased from 7.4 ± 0.7 and 6.1 ± 1.3 preoperatively to 2.7 ± 1.4 and 2.3 ± 0.8 postoperatively, respectively, at the last FU (*P* < .05). NDI scores decreased from 52.7 ± 6.4 preoperatively to 16.3 ± 4.8 postoperatively, at the last FU (*P* < .05) (Table 2).

**Table 2:** Radiographic and clinical outcomes of 64 patients.

	Preop	Latest FU
Disc height (mm)	6.2 ± 0.8	5.8 ± 1.4
C2–C7 Cobb angle (°)	13.5 ± 6.5	14.2 ± 7.1
ROM (°)	37.7 ± 9.6	38.1 ± 8.4
VAS of neck pain	7.7 ± 0.5	3.3 ± 0.9^*^
VAS of radicular arm pain	5.2 ± 1.8	2.2 ± 0.9^*^
JOA scores	14.6 ± 0.7	17.1 ± 0.6^*^
NDI (%)	52.4 ± 6.7	34.8 ± 2.6^*^

Values in data cells are represented as mean ± standard deviation.

FU = follow-up, JOA = Japanese Orthopedic Association, NDI = neck disability index, Preop = preoperative, ROM = range of motion, VAS = visual analog scale.

* Comparing with the preoperative data, *P* < .05.

**Figure 2.**
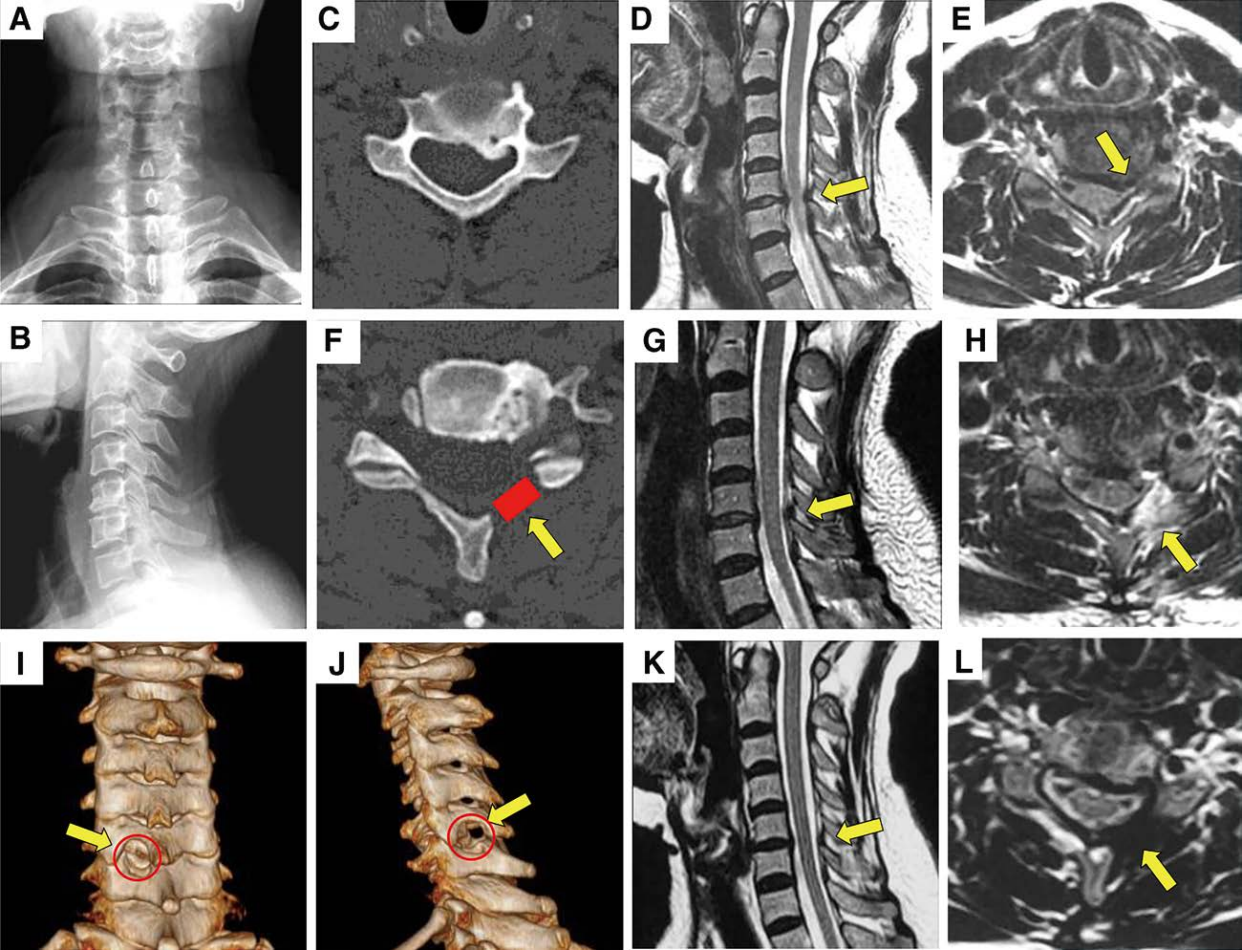
A 48-yr-old female patient with CSR of C5–6 underwent PPECLF. Preoperative radiographic images, including radiographic (A and B), CT (C) MRI (D, E). Postoperative radiographic images, including CT (F), MRI (G and H), CT 3-dimentional reconstruction (I and J). The red zone indicates the range of dorsal decompression. Two year after surgery, the decompression remained unchanged (K and L). CSR = cervical spondylotic radiculopathy, CT = computed tomography, MRI = magnetic resonance imaging, PPECLF = posterior percutaneous endoscopic cervical laminoforaminotomy.

The preoperative results of the radiological evaluation were sustained compared to the postoperative results. Final FU (6.1 ± 1.6 mm) and postoperative (6.2 ± 0.8 mm) disc height changes showed no significant differences. There were no significant differences in the Cobb angle or cervical ROM values at the latest FU compared to preoperative values (*P* > .05) (Table 2). Figure 2 shows a typical case of a patient in this study.

### 3.2. Evaluation of learning curve outcomes

**Figure 3.**
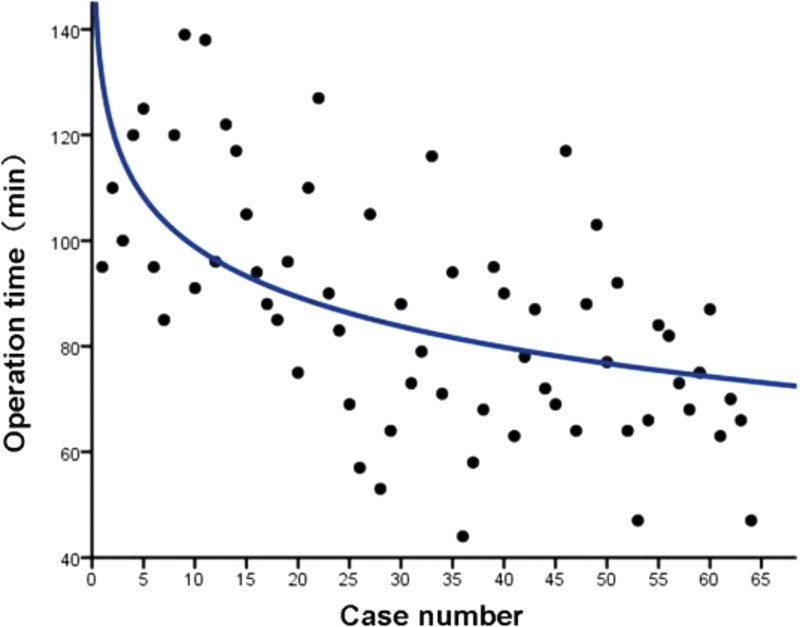
Learning curve of PPECLF as shown by operation time. PPECLF = posterior percutaneous endoscopic cervical laminoforaminotomy.

The logarithmic regression curve (*y* = -13.73ln(*x*) + 130.453, *R*^*2*^ = 0.307) was obtained using bivariate regression analysis of the operation time and number of cases. The mean operative time steadily decreased with the progression of case numbers. After ≈26 patients, the curve plateaued and the operation time was maintained at an average of ≈80 minutes (Fig. 3).

**Figure 4.**
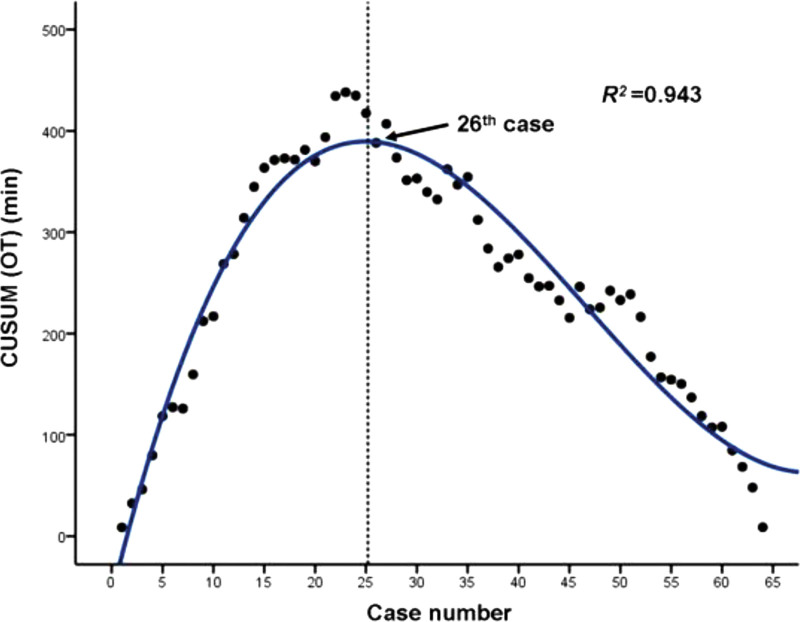
Cumulative sum analysis for operation time of PPECLF. The CUSUM curve for operative time crossed the peak at the 26th case. CUSUM = cumulative sum, PPECLF = posterior percutaneous endoscopic cervical laminoforaminotomy.

The optimal fitting equation of the learning curve (*y* = 0.008 *×* ^*3*^−1.118 *×* ^*2*^ + 40.893*x*−58.776, *R*^*2*^ = 0.943, *P* < .05) was obtained using CUSUM analysis. The curve crossed the peak when the number of accumulated cases reached 26, which was the minimum number of cases needed to enter the mastery phase (Fig. 4).

With the 26th case as the cutoff point, the cases were divided into accumulation (group A) and mastery (group B) phase groups. There were no significant differences in sex, age, symptom duration, operative segment, body mass index, neurological function, or smoking history between the 2 groups (*P* > .05) (Table 3). The operation time in group B was significantly shorter than in group A (*P* < .05). There were no significant differences in the VAS scores for the neck and upper limb, JOA scores, or NDI values between the 2 groups preoperatively or at the latest postoperative FU (*P* > .05). The results of the modified Macnab criteria showed no significant differences between the 2 groups (*P* > .05), and the overall excellent rate was 82.8%. There were no postoperative complications, such as spinal cord nerve injury or incision infection, and no recurrence was observed in either group (Table 4).

**Table 3:** Demographic data of 2 groups divided by the 26th case.

	Group A (No. 1–26)	Group B (No. 27–64)	*P* value
Age (yr)	59.5 ± 8.7	61.9 ± 10.4	>.05
Sex			>.05
Male	12	17	
Female	14	21	
Operative segment			>.05
C3–4	5	8	
C4–5	7	11	
C5–6	12	15	
C6–7	2	4	
BMI (kg/m^2^)			>.05
Normal (18.5–23.9)	14	19	
Overweight (24–27.9)	7	11	
Obese (≥28)	5	8	
Neurologic function			
Motor deficits	11	18	>.05
Sensory deficits	15	20	>.05
Symptoms duration			>.05
<6 mo	14	18	
≥6 mo	12	20	
Tobacco use			>.05
Yes	9	13	
None	17	25	

Values in data cells of age are represented as represent mean ± standard deviation, and the others are represented as number of case. *P* value, comparing group A to group B.

BMI = body mass index.

**Table 4:** Comparison of clinical efficacy between 2 groups.

	Group A (No. 1–26)	Group B (No. 27–64)	*P* value
Operation time (min)	101.2 ± 30.4	77.3 ± 18.9	**<.05**
Blood loss (mL)	17.4 ± 5.5	15.3 ± 3.5	>.05
Postop hospitalization day (d)	14.6 ± 5.1	12.4 ± 5.8	>.05
Hospitalization cost (yuan)	66497.4 ± 6566.3	67943.2 ± 5873.2	>.05
VAS of neck pain			
Preop	7.8 ± 0.9	6.8 ± 0.6	>.05
Latest FU	3.7 ± 0.6	3.9 ± 0.8	>.05
VAS of radicular arm pain			
Preop	6.3 ± 1.1	5.7 ± 1.5	>.05
Latest FU	3.3 ± 1.6	2.9 ± 1.8	>.05
JOA scores			
Preop	15.2 ± 1.2	14.4 ± 0.8	>.05
Latest FU	16.8 ± 0.5	17.3 ± 0.9	>.05
NDI scores (%)			
Preop	53 ± 4.6	51.9 ± 7.7	>.05
Latest FU	16.6 ± 4.4	15.8 ± 5.3	>.05
Complications			
Dural tear	0	0	
Infection	0	0	
Neurodocitis	0	0	
Reoperation	0	0	
Modified Macnab criteria			>.05
Excellent	14	19	
Good	7	13	
Fair	4	6	
Poor	0	0	

Bold value indicates statistical significance. Values in data cells of complications and modified Macnab criteria are represented as number of case, and the others are represented as mean ± standard deviation. *P* value, comparing group A to group B.

FU = follow-up, JOA = Japanese Orthopedic Association, NDI = neck disability index, Preop = preoperative, VAS = visual analog scale.

## 4. Discussion

### 4.1. Clinical efficacy and advantages of PPECLF in CSR

With the development of minimally invasive techniques in recent years, posterior endoscopic cervical laminectomy and discectomy have been widely recognized and gradually performed. These techniques have been shown to be safe and effective in the treatment of CSR, particularly in the treatment of lateral disc herniation or nerve root compression caused by foramen stenosis.^[20]^

The PPECLF technique used in this study was based on endoscopic surgery and traditional anatomy. The nerve root of the responsible segment can be accessed directly through the posterior cervical tissue using a small aperture channel, which is more advantageous than an open surgical approach.^[21]^ The minimally invasive incision avoids extensive dissection of the paravertebral muscles, excessive cutting of the surrounding ligaments, and maximally protects the stability of the cervical spine and the integrity of biomechanical properties.^[22,23]^ The excision range of the facet joint is < 50%, which can better maintain the stress equilibrium of the posterior cervical joint and reduce the incidence of postoperative neck and shoulder axial pain.^[24]^

Ruetten et al^[26]^ compared the effect of percutaneous endoscopic cervical posterior foraminotomy and ACDF in the treatment of CSR and found no significant differences between the 2 surgical methods regarding treatment outcomes, complications, and recurrence rate. In this study, neck and upper limb VAS, JOA, and NDI scores of the patients were significantly improved postoperatively and sustained, thus achieving the same results as those of open approach surgery.

### 4.2. Radiographic outcomes of PPECLF in CSR

The loss of disc height after anterior percutaneous endoscopic cervical discectomy is related to disc destruction during decompression.^[25]^ Ruetten et al^[26]^demonstrated no cervical kyphosis or instability after posterior endoscopic cervical foraminoplasty. There were no obvious changes in postoperative disc height with PPECLF, compared to preoperative values. No significant collapse was observed postoperatively, indicating that PPECLF had little influence on cervical intervertebral discs or surrounding tissues and had obvious advantages in maintaining disc height.

During PPECLF, only the prominent nucleus pulposus and proliferating osteophytes that compressed the nerve root were removed; additionally, the nucleus pulposus was maximally retained, and the nerve root was fully decompressed. This had little effect on the natural mechanical characteristics of cervical vertebrae and reduced the incidence of postoperative ASD, which significantly differed from ACDF and cervical disc replacement. Kim et al^[27]^ demonstrated that within a cohort of 32 patients treated for CSR with posterior percutaneous endoscopic cervical discectomy, no significant postoperative loss of the Cobb angle was observed. In our study, no significant postoperative changes in the Cobb angle or cervical ROM were found, indicating that PPECLF had no significant effect on the segmental or overall stability of cervical vertebrae in mid-term FU.

### 4.3. Learning curve of PPECLF in CSR

In 1954, Ashraf et al^[28]^ first reported CUSUM analysis, which was then introduced into medical clinical analysis in the 1970s. According to this sequential analysis, the change in original data is transformed into the cumulative sum of the difference between each value and the average, which displays the changing trend of the data properly and objectively represents the learning curve. Presently, CUSUM is widely used in the study of learning curves for surgical techniques.^[29,30]^

To date, there is limited data that involves the learning curve of posterior endoscopic cervical laminoforaminotomy in patients with CSR. We herein performed curve fitting for the operation time using bivariate regression analysis, which demonstrated that the operation time was gradually shortened with the increase in the number of cases. The learning curve was plotted based on the CUSUM value and showed that the peak of the learning curve could be achieved after 26 cases. The learning process of PPECLF was divided into 2 stages based on this cutoff point: the accumulation (group A) and mastery (group B) phases, in the early and late stages, respectively.

The operation time in group B was significantly less than that in group A, while there were no significant differences in general information, amount of bleeding, complications, postoperative hospitalization day, or total hospitalization cost between the 2 groups. Additionally, no significant differences were found in the preoperative or postoperative VAS score, JOA score, or NDI value between the 2 groups, which indicated that operation time was the only significant difference in mid-term prognosis between the 2 stages. Therefore, we believe that PPECLF has a significant learning curve and can actively be performed by beginners for the treatment of CSR. Moreover, with the development of navigation and artificial intelligence technology, better preoperative planning can be achieved. We will further use real-time navigation to improve the precision of puncture and decompression, which may significantly shorten the learning curve for enabling precision medicine.

### 4.4. Contraindications of PPECLF in CSR

One of the key prerequisites for the success of minimally invasive techniques is the selection of appropriate cases, and PPECLF has a limited scope of application. Currently, the contraindications include severe cervical spinal stenosis, severe ossification of the posterior longitudinal ligament and cervical instability or deformity, and cervical spondylotic myelopathy caused by anterior compression. However, in recent years, some scholars have performed endoscopic decompression for the treatment of cervical spondylotic myelopathy and achieved good results.^[31–33]^ The author believes that with the progress of optical technology and innovation of instruments, the indications for PPECLF will gradually expand.^[34]^

### 4.5. Study limitations

Due to the small sample size, short FU time, and lack of a control group of surgeons, this study demonstrated a limited analysis of the PPECLF technique. Therefore, it needs to be further confirmed by accumulated cases and long-term FU. In addition, the cervical spine function scores used in the study were widely used in traditional open approach surgery, and whether they are fully applicable to minimally invasive techniques requires further research.^[35]^

## 5. Conclusions

PPECLF is based on endoscopic surgery and the traditional anatomical path and can achieve the same clinical efficacy as that of open approach surgery. PPECLF has a significant learning curve in the treatment of single-segment CSR. It takes 26 cases for beginners to reach the mastery phase; however, a longer operation time has no impact on the previous mid-term clinical outcome.

## Acknowledgments

The authors are grateful to Yuling Wang for the clinical images, data collection, and patient follow-up.

## Author contributions

Conceptualization: Ran Yao, Ming Yan, Fenglong Sun.

Data curation: Qingchen Liang, Ke Li.

Formal analysis: Ran Yao, Ming Yan.

Investigation: Ran Yao, Ming Yan.

Methodology: Ran Yao.

Project administration: Ran Yao, Fenglong Sun.

Resources: Ran Yao.

Software: Ran Yao.

Supervision: Hongqing Wang, Zuyao Liu, Fu Li, Hao Zhang.

Validation: Qingchen Liang, Ke Li.

Visualization: Ran Yao.

Writing - original draft: Ran Yao.

Writing - review & editing: Ran Yao, Fenglong Sun.

## Supplementary Material


